# Supplementary material for: Multi‐dataset identification of innovative feature genes and molecular mechanisms in keratoconus
Source: J Cell Mol Med. 2024 Sep 19;28(18):e70079. doi: 10.1111/jcmm.70079 (PMC11412914; doi:10.1111/jcmm.70079)
Supplement: Supplementary file 2 — Material S2. [file JCMM-28-e70079-s002.zip › 7.GSEA analysis/6.SNORA74B/KEGG_ETHER_LIPID_METABOLISM.html]

Details for gene set KEGG\_ETHER\_LIPID\_METABOLISM[GSEA]

|  || Dataset | SNORA74B.group.cls#h\_versus\_l |
| Phenotype | group.cls#h\_versus\_l |
| Upregulated in class | l |
| GeneSet | KEGG\_ETHER\_LIPID\_METABOLISM |
| Enrichment Score (ES) | -0.61085695 |
| Normalized Enrichment Score (NES) | -1.5381867 |
| Nominal p-value | 0.035856575 |
| FDR q-value | 0.31687337 |
| FWER p-Value | 0.716 |
Table: GSEA Results Summary

  

Fig 1: Enrichment plot: KEGG\_ETHER\_LIPID\_METABOLISM      
 Profile of the Running ES Score & Positions of GeneSet Members on the Rank Ordered List

  

| SYMBOL | TITLE | RANK IN GENE LIST | RANK METRIC SCORE | RUNNING ES | CORE ENRICHMENT || 1 | PLA2G4E | na | 5383 | 0.212 | -0.1077 | No |
| 2 | PLA2G12B | na | 10125 | 0.090 | -0.2242 | No |
| 3 | PLA2G10 | na | 10767 | 0.079 | -0.2251 | No |
| 4 | JMJD7-PLA2G4B | na | 13953 | 0.036 | -0.3088 | No |
| 5 | AGPS | na | 16078 | 0.014 | -0.3669 | No |
| 6 | PLA2G12A | na | 16297 | 0.012 | -0.3706 | No |
| 7 | PLA2G6 | na | 16344 | 0.011 | -0.3694 | No |
| 8 | PLD2 | na | 16641 | 0.008 | -0.3762 | No |
| 9 | PLA2G2F | na | 17001 | 0.005 | -0.3855 | No |
| 10 | PAFAH1B1 | na | 17010 | 0.005 | -0.3847 | No |
| 11 | LPCAT4 | na | 17678 | -0.002 | -0.4033 | No |
| 12 | PLD1 | na | 18510 | -0.012 | -0.4246 | No |
| 13 | PLA2G2E | na | 18608 | -0.013 | -0.4246 | No |
| 14 | PLA2G2D | na | 19469 | -0.023 | -0.4443 | No |
| 15 | PAFAH2 | na | 19525 | -0.023 | -0.4407 | No |
| 16 | ENPP6 | na | 19810 | -0.026 | -0.4430 | No |
| 17 | PAFAH1B3 | na | 20454 | -0.034 | -0.4540 | No |
| 18 | PLA2G4A | na | 24085 | -0.082 | -0.5404 | No |
| 19 | ENPP2 | na | 25199 | -0.102 | -0.5497 | No |
| 20 | PLA2G2C | na | 26678 | -0.132 | -0.5628 | Yes |
| 21 | PAFAH1B2 | na | 26972 | -0.139 | -0.5402 | Yes |
| 22 | PLA2G3 | na | 27299 | -0.147 | -0.5168 | Yes |
| 23 | PLA2G5 | na | 30479 | -0.245 | -0.5538 | Yes |
| 24 | LPCAT2 | na | 32460 | -0.351 | -0.5324 | Yes |
| 25 | PLA2G7 | na | 32748 | -0.372 | -0.4576 | Yes |
| 26 | LPCAT1 | na | 33954 | -0.524 | -0.3753 | Yes |
| 27 | PLA2G2A | na | 33973 | -0.527 | -0.2581 | Yes |
| 28 | PLA2G1B | na | 34211 | -0.585 | -0.1344 | Yes |
| 29 | CHPT1 | na | 34442 | -0.668 | 0.0082 | Yes |
Table: GSEA details [plain text format]

  

Fig 2: KEGG\_ETHER\_LIPID\_METABOLISM      
 Blue-Pink O' Gram in the Space of the Analyzed GeneSet

  

Fig 3: KEGG\_ETHER\_LIPID\_METABOLISM: Random ES distribution      
 Gene set null distribution of ES for **KEGG\_ETHER\_LIPID\_METABOLISM**
